# Costs of diabetes and its complications in Poland

**DOI:** 10.1007/s10198-013-0513-0

**Published:** 2013-07-03

**Authors:** Joanna Leśniowska, Agata Schubert, Michał Wojna, Iwona Skrzekowska-Baran, Marta Fedyna

**Affiliations:** 1 Koźminski University, 57/59 Jagiellońska St., 03-301 Warsaw, Poland; 2 Novo Nordisk, 17 stycznia 45 St., 02-146 Warsaw, Poland; 3 Starowiślna 17/3 St., 31-038 Kraków, Poland

**Keywords:** Diabetes type, Cost of disease, Complications, Indirect cost, H51, I15, I18

## Abstract

**Objective:**

Diabetes mellitus (DM) is a major health problem with severe complications and a significant impact on quality of life. It constitutes an enormous burden of disease due to high prevalence, severe co-morbidities and high costs for society. This study is the first comprehensive study on the direct and indirect costs of DM (type 1 and type 2) and associated complications in Poland.

**Methods:**

In order to estimate the direct medical costs of DM and its complications, including the costs of medical consultation, hospitalisation, rehabilitation, drugs and medical equipment, data from the National Health Fund were used. Indirect costs on loss of productivity due to diabetes and its complications were based on data obtained from the ZUS (Social Insurance Institution) and from GUS (Poland’s Central Statistical Office). Attributable risk methodology was used to assess the burden of DM complications.

**Results:**

A continuous increase of the direct costs of diabetes has been observed since the year 2005. In the analysed time period (2005–2009) the direct costs of medical services for both types of DM doubled. DM is a cause of significant sickness absence and incapacity for work and therefore is associated with a growing productivity decline in Poland. The highest direct costs and indirect costs are associated with treatment of diabetes-related complications. Direct costs of hospital complication treatment were EUR 332 million, which exceeded by more than five times the direct costs of hospital treatment of diabetes per se, which in the same year amounted to EUR 58.5 million. The indirect costs of diabetes-related complications were higher by 41 % compared with indirect costs related to DM itself. Total costs of health care services for DM and its complications amounted to EUR 654 million, which constitutes a 2.8 % of total health care costs in Poland. Total DM cost in Poland in 2009 amounted EURO 1.5 billion.

**Conclusions:**

DM is causing a growing economic burden on the health care system and on Polish society in terms of health care and productivity losses. Most of the total cost of diabetes are indirect costs caused by productivity losses. Both direct and indirect costs are driven by the cost of diabetes complications.

## Introduction

Diabetes mellitus (DM) is a widespread clinical and public health problem in many countries in the world, especially when we take into account its high prevalence, increasing morbidity and impact on health care systems in terms of costs. An estimation of the influence of DM on health care expenditures is complicated because of the many aspects of the issue. Total costs can be divided into medical and non-medical or direct and indirect costs. In addition to costs associated with DM treatment (costs of insulin and antidiabetic drugs, hypoglycaemia hospitalisation, etc.), treatment of immediate and distant future complications caused by DM should be taken into consideration. People suffering from the disease are at high risk of serious cardio- or cerebrovascular problems, neuropathy and/or microvascular complications (nephropathy, retinopathy), which lead to a rise in the total costs of the illness and significantly lower the quality of life. Increased risk of disability, inability to work or premature death is important as well. Loss of productivity caused directly by DM as well as by DM-related complications constitutes a substantial economic burden.

The economic impact of DM on a health care system depends, in particular, on the type of illness. According to the classification recommended by the WHO, there are two main types of DM: type 1 DM and type 2 DM. Although these separate conditions have similar long-term consequences, they affect different age groups and require different treatment strategies. Type 1 DM, previously called insulin dependent, usually develops in childhood and adolescence as a result of impairment of pancreatic islets β-cells, whereas type 2 DM results from insulin resistance—an inappropriate response of body tissue to insulin—and usually develops in adulthood. It is estimated that more than 90 % of cases are type 1 or 2 DM [1, 2]. It is estimated that in Poland approximately 2.5 ml people have DM (6.54 % of the general population), of whom 750,000 are yet to be diagnosed [3]. This high prevalence will have devastating economic and social effects over the medium and longer term.

A number of pharmacoeconomic or cost-of-illness analyses related to DM have been performed recently. However, reported costs vary significantly depending on the data source used, type of assessment and scope of the analysis. Some cost estimates are made on the basis of the existing population data on health care use, disability and mortality, while others are performed using the data of patients with DM at an individual level. Methodologies differ in terms of methods of calculating the direct and indirect costs, costs attributable to DM, and in the way in which the results are presented. Furthermore, a number of investigations are concentrated on one type of DM only. Therefore, the results of different studies either cannot be compared or the comparison is problematic and unreliable.

An analysis performed in Spain [4], conducted in a sample of 517 patients from endocrinology clinics, suffering type 2 DM, showed an annual cost of EUR 4,278 per patient. Direct costs, including, e.g., medication, specialist consultations and hospitalisations, accounted for 58 % of the total costs and were appreciably higher than indirect costs, including temporary or permanent disability and working hours lost. In the costs of DM study (CoDiM) [5], data were collected by the local statutory health insurance fund and the Association of Statutory Health Insurance Physicians in the federal state of Hesse in Germany. It was estimated that more than half of the medical costs per patient with DM in Germany in 2001 (EUR 4,457) was spent on the treatment of DM complications (EUR 2,380), including microvascular complications, foot complications, macrovascular complications and uncontrolled glucose metabolism. In a recent article [6], it was shown that DM costs approximately £23.7 billion in the UK and indirect costs accounted for the majority of the costs (58 %). The study used a top-down approach, estimated the direct and indirect cost of type 1 and type 2 DM and provided cost estimates for 2035/2036. A similar approach was used in a study conducted in Sweden [7]. The study followed an attributed risk methodology to estimate the share of costs directly and indirectly attributed to DM. The estimated cost for Sweden was 920 million euros in 2005 and DM per se accounted for 50 % of the health care cost. Comparisons of the above-mentioned studies have to be made very cautiously because of methodological inconsistency.

So far, three studies of costs related to DM have been conducted in Poland. In the cost of DM type 2 in Poland study (CODIP) [8], direct and indirect costs of type 2 DM were analysed. It has been estimated that the average direct costs per patient with type 2 DM are EUR 539.88, whereas indirect costs are at a level of EUR 1,510.50. This means that in Poland in 2002, direct costs related to type 2 DM accounted for 26 % of the total costs of the illness. It has been shown that the direct costs of DM accounted for 8.08 % of total health care expenditures in Poland. CODIP was designed as a multicentre, retrospective, bottom-up study, based on health insurance data from Mazowieckie province. According to another bottom-up study, the costs of treatment of DM complications in Poland in 2002 exceeded 96.6 million euros [9]. In the third study [10], only the indirect costs of DM were analysed. According to this paper, the average indirect costs of treatment of one patient with DM amount to 1,273 EUR. None of the aforementioned studies provided a comprehensive view of DM costs, which include direct and indirect costs of both DM and its complications. Moreover, up to now there has been no top-down study in Poland to provide estimates based on aggregated data.

Severe criticism has been raised against cost of illness studies [11], pointing out flaws in methodological assumptions and questioning the utility of these analyses for decision makers. Discussion on general methodological issues of COI analyses is not the subject of this article. Despite of all these controversies, the authors believe that the results of this analysis can enhance the process of health care priority setting and allocation of resources in Poland. Furthermore, the results of the study can be a source of cost comparison of different approaches to the management of the disease. Common cost effectiveness and budget impact analyses conducted in Poland do not deliver essential information, as these are usually conducted for a single intervention not a disease management and focus only on the payer perspective.

The goal of this article is to present the results of the first Polish top-down study that aimed to assess the total costs of DM in Poland, including both direct and indirect costs of DM and its complications.

There is a need of a comprehensive and systematic study of the DM-related costs in Poland, as well as discussion on proper DM prevention and management. The number of people living with DM in Poland is expected to increase by 28 % by 2030 as a result of the obesity epidemic, the ageing of the population and other factors such as lifestyle changes and models of consumption. There is great public concern about the obesity epidemic among children in Poland. In the future, these children will face a substantial risk of DM, and this will result in an increasing social and economic burden. Another rationale for our study is to estimate the structure of DM-related costs and to compare them with the results of analyses conducted in other countries. Our results can serve as a benchmark for future studies on the effectiveness of DM management and efficiency of health resources allocation in the health care system and in the public finance sector.

## Materials and methods

### Method

This study is a prevalence-based top-down cost of illness study, which analysed the direct and indirect costs of DM and DM-related complications. We defined prevalence as all patients suffering from DM and alive on 31 December 2009. The resources used for DM per se were identified based on DM being the first diagnosis for resource consumption. Additionally, we estimated the costs of DM complications. DM-related complications included in the analysis were identified based on the ICD-10 codes of complications included in the German CoDiM Study [9]. They are as follows:

- Ischaemic heart diseases: angina pectoris (I20), acute myocardial infarction (I21), chronic ischaemic heart disease (I25)
- Other forms of heart disease: heart failure (I50)
- Cerebrovascular diseases: intracerebral haemorrhage (I61), cerebral infarction (I63), stroke, not specified as haemorrhage or infarction (I64)
- Renal failure (N17–N19)
- Retinal disorders in diseases classified elsewhere (H36)
- Visual disturbances and blindness: visual impairment including blindness (H54).

The research was based on the data acquired from the National Health Fund (NFZ), ZUS (Social Insurance Institution), and from GUS (Poland’s Central Statistical Office).

### Direct costs

The direct costs assessed in the research included: medical care (i.e. outpatient consultations, hospitalisation) and drug reimbursement calculated from the payer perspective. Due to the marginal share of co-payments in the public health care system the costs calculated from the payer perspective are a good estimate of social cost.

Intangible costs have been omitted. The scope of the analysis does not include the costs associated with indirect costs of informal care, which can account for up to 30 % of the indirect costs in DM treatment [6]. The cost of medical care (i.e., medical consultations, hospitalisation) related to type 1 and 2 DM was determined on the basis of the data acquired from the NFZ. The costs of medical services rendered within the system of Primary Health Care (PHC) were calculated based on a report published by NFZ. Both sources include expenditures incurred by health care providers. The cost analysis of drug reimbursement was based on the data included in NFZ reports available on the Fund’s homepage. The analysed costs of drug reimbursement include the costs of oral antidiabetics and insulin.

A calculation of direct costs of DM-related complications was also carried out on the basis of the data acquired from NFZ. These data cover complete information about all patients treated in hospitals in 2009. The data were extracted by NFZ in two stages. First, patients with a diagnosis of primary and co-morbid DM (i.e. E10, E10.0–E10.9, E11 and E11.0–E11.9) were selected. Then, episodes containing primary or DM-related co-morbidity were identified in these patients. The direct costs of DM complications were calculated using aetiological fractions (EF), to estimate what share of co-morbidity costs is attributable to DM. The EF is calculated based on the following:

$$ {\text{EF}} = \frac{{P \left( {R - 1} \right)}}{{P\left( {R - 1} \right) + 1}} $$

where *P* stands for the prevalence rate of DM among hospital patients, and *R* is a relative risk of suffering from a given chronic complication among people with DM [12].

The methodology applied is a source of possible systematic error in our estimates. When calculating relative risks and prevalence rates values we used panel data of hospital patients as a sample. This sample is not representative of the entire population with overrepresentation of patients with severe DM; therefore there is a risk that the estimated variables are overestimated. This possible bias can thus lead to overestimation of the indirect costs share in the total costs. Moreover, because of this limitation in methodology, the calculated values of EFs are not easily comparable with the results of other epidemiological studies.

### Indirect costs

The indirect costs assessed in the research included the costs of productivity loss due to work absence or inability to work due to DM and DM complications. The indirect costs were estimated using the data from ZUS to GUS. In the case of the indirect costs of DM-related complications, similarly to the approach applied in the direct cost estimations, we used EF to estimate what share of the total indirect cost of chosen co-morbidities can be attributed to DM. ZUS reports the first diagnosis in the ICD-10 coding; therefore it was impossible to directly identify patients who were unable to work because of DM-related complications.

While estimating the indirect costs, the human capital method was used. The costs of lost productivity due to sickness absence were calculated with the use of the ZUS data (i.e. the number of days taken off for sickness caused by type 1 and 2 DM) and the average daily gross wage in the economy (EUR 34.06 in 2009). The analysis of indirect costs also involved lost productivity related to work incapacity. These costs are incurred because of inactivity in the labour market and the collection of social security benefits and were calculated on the basis of the ZUS data concerning the volume of social security benefits transferred, along with the GUS data on the average gross salary in the economy.

Lost productivity caused by premature death was not included in the analysis of indirect costs because of the lack of data.

### Sensitivity analysis

In our study we applied point estimates of EF for DM complications. The variables used to calculate EF are subject to uncertainty; therefore we provided upper and lower limits of the direct and indirect costs resulting from adjusting the relative risks and prevalence rate. We applied sensitivity analysis of ±10 % to relative risks. With regard to the prevalence rate, we used the results of an epidemiological study conducted in the Halemba district in Poland (6.54 %) [3] and the data provided by the International DM Federation (10.54 %) as the lower and upper bound respectively [13].

## Results

### Direct costs: hospitalisation and outpatient care

**Fig. 1:**
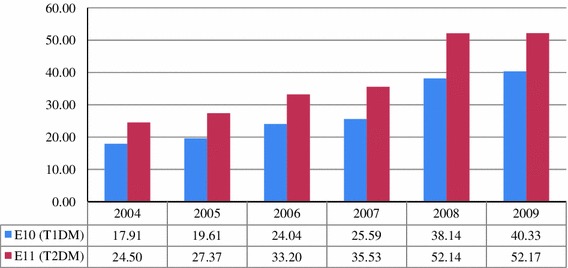
The costs DM services (without the costs of drugs and PHC) in 2004–2009 (in million euros)

Figure 1 shows the costs of health care services provided in 2004–2009 related to type 1 and type 2 DM. In 2004–2009 a significant increase in the costs of health care services provided in relation to type 1 and type 2 DM was observed. In the case of type 1 DM the rate of increase in the costs was 125 %, and for type 2 DM it was 129 %.

In the DM services analysis, care of patients treated within the PHC network was not included because of the method of reporting by the NFZ. Estimation of the PHC costs was, however, possible for 2009. In 2009, care for patients with DM was granted a higher financing level. General practitioners treating DM patients receive a yearly lump sum assigned to an individual patient that is three times higher than that for a ‘regular’ patient. The data from the performance of the contracts with the PHC doctors show that the total number of patients with DM who were treated in POZ in 2009 was 1,584,716. Thus, we can estimate the PHC costs of DM treatment in 2009 to be EUR 70.4 million.

The total costs of health care services for type 1 and type 2 DM in 2009 amounted to EUR 163 million, and the largest individual factors are the costs of PHC and hospital treatment, which constitute 82.8 % of the total costs.

### Direct costs: drug reimbursement

On the basis of the available data there is no possibility of classifying the costs of drugs according to the type of DM. Therefore, the costs were analysed for both DM types. The total costs of drug reimbursement for DM in 2009 amounted to EUR 159 million. The distribution of the costs for the years 2005–2009 demonstrated that the total expenditures for drugs have increased by 25.7 % since 2005.

### Total direct costs

The available data do not allow for a separate analysis of the drug reimbursement and PHC costs for each type of DM. Therefore the total direct costs were calculated together for type 1 and type 2 DM (Table 1) and they amounted to EUR 322 million. The largest shares of the direct costs are drug reimbursement, PHC and hospital treatment, which are respectively 49, 22 and 18 %.

**Table 1:** The values of total direct costs related to diabetes in Poland in 2009 (in EUR mln)

	Type 1 DM + type 2 DM	
	Total	Per 100,000
Direct—total	322.03	0.84
Hospital treatment	58.51	0.15
Drugs reimbursement	159.08	0.41
GPs	70.43	0.18
Heath services—without drugs and GPs	92.51	0.24

### Direct costs of complications

Since 2009, the NFZ database has made it possible to extract the costs of hospital treatment for DM-related complications. Calculated EFs for people diagnosed with DM (in hospital treatment) are presented in Table 2.

**Table 2:** Calculated attributable risks for diabetes-related chronic complications in Poland in 2009 (in hospital treatment)

Diseases	EF
Angina pectoris (I20)	0.41
Acute myocardial infarction (I21)	0.40
Chronic ischaemic heart disease (I25)	0.39
Heart failure (I50)	0.69
Intracerebral haemorrhage (I61)	0.21
Cerebral infarction (I63)	0.40
Stroke, not specified as haemorrhage or infarction (I64)	0.18
Acute renal failure (N17)	0.19
Chronic kidney disease (N18)	0.20
Unspecified kidney failure (N19)	0.19
Retinal disorders in diseases classified elsewhere (H36)	0.27
Visual impairment including blindness (H54)	0.14

In 2009, the direct costs of hospital complication treatment were EUR 332 million, which exceeded by more than five times the direct costs of hospital treatment of DM per se, which in the same year amounted to EUR 58.5 million. The biggest share in the costs of DM-related complications goes to heart diseases (84.4 %).

### Indirect costs of DM

The total indirect costs of type 1 and type 2 DM consist of the following fractional costs: the costs of lost productivity because of sickness absence and the costs of lost productivity due to work incapacity.

**Fig. 2:**
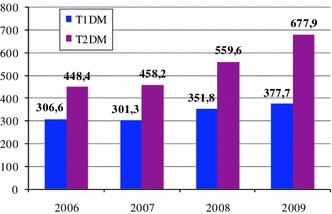
The number of sickness absence days due to type 1 DM (T1DM) and type 2 DM (T2DM) in thousands

The costs of lost productivity because of sickness absence in the case of type 1 DM were EUR 12.8 million and in the case of type 2 DM were EUR 32.1 million. The analysis of absenteeism revealed a growing trend of indirect costs related to both types of DM in the period 2006–2009. A 74 % growth in the costs was observed for both types of DM; however, a more rapid increase in the costs was observed for type 2 DM patients (88.6 vs. 53.7 %). This correlated with an increase in the number of sickness absence days by 23.2 % for type 1 DM and 51.2 % for type 2 DM (Fig. 2).

Lost productivity due to work incapacity in the case of type 1 and type 2 DM was respectively EUR 223.6 million and EUR 79.5 million.

In 2009, the total indirect costs of DM exceeded EUR 349 million, of which around 13 % was associated with loss of productivity because of sickness absence, whereas the remaining 87 % with lost productivity was attributed to work incapacity.

### Indirect costs of complications

The indirect costs of complications include the costs of lost productivity caused by sickness absence and costs of lost productivity related to work incapacity. These costs were EUR 53.8 million and EUR 438.05 million respectively. The structure of indirect costs related to DM-related complications was then similar to the corresponding cost structure of DM itself and demonstrated that 89 % of the costs were associated with productivity losses due to incapacity for work, while only 11 % with capacity lost because of sickness absence days.

In both the costs of lost productivity due to sickness absence and the costs of lost productivity related to work incapacity, the prevalence of heart disease complications was respectively 91.5 and 83.9 %.

Overall, the indirect costs of DM-related complications were higher by 41 % compared with the indirect costs related to DM itself.

### Total costs of DM and complications in 2009

**Fig. 3:**
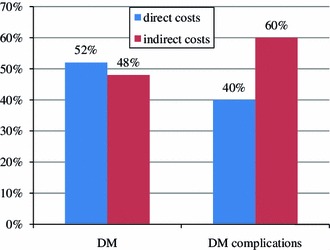
Direct and indirect costs of diabetes and its complications related to the total costs

The total costs of DM in 2009 amounted to EUR 1.5 billion (Table 3). In the structure of the total costs of DM and its complications, there is a prevalence of indirect costs (56 %). Figure 3 shows that in the structure of the total costs of DM there is a slight prevalence of direct costs (52 % of total costs). In the case of costs of complications there is a domination of indirect costs (60 % of total costs).

**Table 3:** The values of costs related to diabetes and its complications in Poland in 2009 (in million euros)

	Type 1 DM + type 2 DM		Diabetes complications		Total cost of diabetes	
	Total	Per 100,000	Total	Per 100,000	Total	Per 100,000
Direct—total	322.03	0.84	332.24	0.87	654.27	1.71
Heath services—without drugs and GPs	92.51	0.24	332.24	0.87	424.75	1.11
Drugs reimbursement	159.08	0.41	NA	NA	159.08	0.42
GPs	70.43	0.18	NA	NA	70.43	0.18
Indirect—total	349.01	0.91	491.89	1.28	840.90	2.20
Loss productivity due to sickness absence	45.96	0.12	53.83	0.14	99.79	0.26
Loss productivity due to incapacity for work	303.05	0.79	438.05	1.14	741.11	1.94
Total without drugs reimbursement and GPs	441.53	1.15	824.13	2.16	1,265.66	3.31
Total	671.04	1.75	824.13	2.16	1,495.18	3.91

### Sensitivity analysis

A sensitivity analysis (Tables 4, 5) revealed low elasticity of both direct and indirect costs with respect to relative risks and prevalence rate. This is mainly attributed to the very high absolute values of estimated EFs.

**Table 4:** Sensitivity analysis for DM direct costs

Variable	Sensitivity: lower value	Model value	Sensitivity: upper value
Relative risk	674,285,493.47	689,490,239.73	712,668,739.07
Prevalence rate	674,478,036.72	689,490,239.73	763,274,239.59

**Table 5:** Sensitivity analysis for DM indirect costs

Variable	Sensitivity: lower value	Model value	Sensitivity: upper value
Relative risk	795,148,021.56	830,909,121.87	859,814,374.69
Prevalence rate	796,725,482.51	830,909,121.87	943,988,689.14

The analysis shows that a 10 % increase in relative risks results in a 3 and 3.4 % increase in the direct and indirect costs respectively. The values of the direct and indirect costs calculated for lower limits of relative risks are 2.3 and 4.4 % lower in comparison with the model values. The figures in Tables 5 and 6 also reveal low elasticity of the direct and indirect costs with respect to the changes in the prevalence rate values.

**Table 6:** Average cost of medical services (without PHC and drug reimbursement) provided in relation to the type 1 DM and type 2 DM in 2004-2009 (in EUR)

	Years					
	2004	2005	2006	2007	2008	2009
Type 1 DM	112.69	128.53	126.99	148.12	209.25	214.49
Type 2 DM	70.28	77.33	71.33	81.48	103.58	95.74

## Conclusions

From a social perspective, the total annual cost of DM in Poland is about EUR 1.5 billion. The total costs of health care services for DM and its complications amount to EUR 654 million, which constitutes 2.8 % of the total health care costs in Poland. Since 2005 a systematic increase in direct costs of DM treatment has been observed. In the case of type 1 DM such a substantial increase in the costs was caused by a more than 90 % rise in the average cost of medical services (Table 6). This was caused mainly by the reimbursement of insulin pumps and related accessories for children, which was first undertaken at that time. The increase in the costs of type 2 DM was associated with an increase of epidemiological indicators, because the average cost of medical services in 2009 increased by 36 % as compared to 2004. The highest costs are connected with the treatment of DM-related complications, which are more than five times greater than the costs of DM treatment (including only hospital care); these results correspond to the characteristics observed in other European studies. It confirms that the treatment priorities adopted by the European and Polish DM Associations, which recommend treatments to target and result in a decreasing incidence of DM-related complications, are reasonable not only from a clinical point of view but can also generate cost savings. Lower incidences of DM-related complications will have a major impact on the total cost of DM, and this is a way to halt the economic burden of DM. The study shows that a significant share of the total costs (56 %) of DM and its complications is constituted by indirect costs. The indirect costs of DM are often overlooked in health care planning in Poland; the results of this study show that indirect costs can be higher than direct costs and therefore cannot be ignored in health care decision-making processes. The usage of health care resources should be planned not only to cut direct costs of treatment but also to consider the social impact of the disease, and through effective treatment to minimise indirect costs. Sickness absence and work incapacity can largely diminish productivity when the disease is not managed correctly.

This is the first time that the attributable risk approach has been used for estimating the direct and indirect cost of DM complication in Poland. The Polish DM COI studies mentioned above have estimated the cost of DM complications directly from the study sample without adjusting for attributable risk. Due to a major methodology discrepancy the results are barely comparable with the previous studies conducted in Poland, although it can be observed that indirect costs constitute a high fraction of the total costs regardless of the methodology used.

The fact that both the total health care cost and the cost per person diagnosed with DM were found to be lower than in the UK [6] is not surprising as the cost of resources and the number of people with DM are much lower in Poland. There is also a substantial difference in the cost compounds proportion; in this characteristic Poland is more similar to Sweden. Our estimate of the health care cost as a result of DM per se was very similar to the estimate of Bolin et. al [7]. DM per se in Poland accounted for 49 % of the health care cost and 41 % of the productivity loss; the corresponding figures in Sweden were 50 and 41 % respectively. Despite these similarities DM in Poland seems to constitute a more severe burden to the health care budget. In Sweden DM-related health care costs accounted only for approximately 1.4 % of the total health care cost; in Poland this fraction was more than double (2.87 %). Polish DM patients seem to be more severe cases; our study reported almost twice as many hospitalisations per 100,000 inhabitants than in Sweden and hospital costs in Poland are more than 50 % lower (Table 7). In comparison to the mentioned Western European countries, our study results report substantially lower estimates for DM cost per capita. It would be desirable to compare the Polish results to the estimates from other Eastern European countries.

**Table 7:** Hospital care costs of diabetes and diabetes-related chronic complications in Poland 2009, total and per 100,000 inhabitants (costs in EUR million)

	DM		DM complications		DM and DM complications	
	Total	Per 100,000	Total	Per 100,000	Total	Per 100,000
Hospital care						
Number of hospital admissions	103,328	270.72	341,292	894.20	444,620	1,164.93
Costs	58.51	0.15	332.24	0.87	390.75	1.02

Most of the previous studies in Poland were based on epidemiological data and surveys conducted on a representative sample of the Polish population. This results from low data availability, dispersion or a lack of data. Our top-down study of DM costs is the first of its type in Poland. It is based on reliable source data, and although some data could not be obtained, the results provide an insight into the structure of DM costs while demonstrating the scale of the problem. Such measurements are the first step in the proper DM control, and this study can be a starting point for further research in this field in Poland to provide a full picture of the DM burden. An improvement of data availability and quality is needed to monitor the real costs of the disease. We should indicate a number of difficulties connected with conducting COI studies in Poland. These are primarily low data availability, dispersion or lack of data: difficulty in identification of costs related to the most frequent complications, lack of legal regulations and underdevelopment of the information infrastructure of the health care system.

The study has certain limitations that should be mentioned. It is likely that the results underestimate the real costs associated with DM and DM-related complications. One reason is not including the loss of productivity associated with mortality. This cost can account for a substantial part of the total DM cost. A recent DM COI study reported that it can amount to as much as 46 % of the total indirect DM burden [14]. The study does not include information on direct costs of long-term care in formal care centres or indirect costs of informal care provided to disabled DM patients. It can be expected that the second group of costs would constitute a significant share in Poland. Informal care is the most common form of providing long-term care in Poland. There is also a limitation connected with estimation of relative risks used for calculation of costs of complications attributable to DM. There is no DM registry in Poland; therefore calculations were based on hospital data. DM patients treated in hospitals tend to have more severe diseases in comparison to a general sample of DM patients, as not all DM patients have to be treated in the hospital. This inevitably leads to problem with confounding variables and results in upward bias in relative risks and prevalence rate values. However, strikingly high values for some of the relative risks potentially can also comprise some information about the effectiveness of the management of chronic conditions in the Polish Health Care System. This general issue is beyond the scope of our analysis, but we believe it deserves attention in future COI-related studies.

In conclusion, our study shows that DM imposes a major economic burden in Poland. Therefore there is a need to introduce an integrated system of DM management that would enable containing future DM costs.
